# Quality of health care for refugees – a systematic review

**DOI:** 10.1186/s12914-019-0205-7

**Published:** 2019-06-13

**Authors:** Karolin Hahn, Jost Steinhäuser, Denise Wilfling, Katja Goetz

**Affiliations:** Institute of Family Medicine, University Hospital Schleswig-Holstein, Campus Luebeck, Ratzeburger Allee 160, 23538 Luebeck, Germany

**Keywords:** Quality of health care, Quality indicators, Health care, Refugees, Review

## Abstract

**Objective:**

The aim of this systematic review was to identify quality indicators (QI) developed for health care for refugees.

**Methods:**

We conducted a systematic review of international QI databases such as the Agency for Health care Research and Quality in addition to a systematic search in PubMed, Cochrane library and Web of Science, using the terms “refugee” and “quality indicator”, complemented by a search in reference lists and grey literature. All papers which included QIs for refugees, especially for health care were included. In a first step all existing QIs were screened for their relevance to refugees. In a second step, all health care QIs were extracted. In a final step, these health care QIs were classified into process, structure and outcome indicators.

**Results:**

Of 474 papers, 23 were selected for a full-text review. Of these 23 publications, 6 contained 115 QIs for health and health care for refugees. The main health care topics identified were reproductive health, health care service and health status.

**Conclusions:**

Most indicators were indicators for outcome and structure quality, the smallest group were process indicators. Within the area of refugee health care, most QIs that have been found were QIs regarding reproductive health. QI databases do not yet include indicators specifically related to refugees.

## Background

Health care for refugees and asylum seekers represents a challenge for the health system of the host country for various reasons. Examples are lack of access to health care in the host country and past experience of trauma which may have caused mental health problems. Furthermore, there may be barriers of communication, language and culture [1–3]. As a result of persecution, conflict, generalized violence or human rights violations, 65.3 million people were forcibly displaced worldwide in 2015 [4]. Most refugees were from Syria and over 6.3 million people fled from war [4].

Back in 1995, the UN High Commissioner for Refugees (UNHCR) highlighted the ‘urgent need to address the areas of safe motherhood, control of HIV/AIDS/STD, family planning services, and management of sexual and gender based violence within the overall primary health care services’ [5]. However, the assurance of a good quality of health care for refugees and asylum seekers will be more and more important for the health system in the host countries.

A core dimension of health system performance is health care quality [6]. Quality of care can be defined as ‘whether individuals can access the health structures and processes of care which they need and whether the care received is effective’ [7]. Furthermore, quality of care should be divided into three dimensions: structure, process and outcome of care, which could result in measureable quality indicators (QI) [8]. QIs are important for the assessment of health care and are essential measurement tools for documentation and improvement of quality of care [9]. Measurable QIs for health care for refugees have not been identified until now. It can be assumed that regular QIs are just as valid for refugees as they are for all other patients. However, there are specific refugee situations like health care in refugee camps for which there should be quality assurance too. Therefore, the aims of this systematic review were to evaluate and to extract QIs developed for refugees and asylum seekers.

## Methods

### Systematic review

This systematic review was conducted to find existing QIs, concentrating on those relevant for refugee care, as there are some specific requirements in a typical “refugee situation” as in humanitarian crisis situations, refugee camps, reception centres and health care for refugees and asylum seekers in host countries. Different international and national indicator databases were screened in June 2018. These databases were: the Agency for Health care Research and Quality (AHRQ), the UK’s Quality and Outcomes Framework (QOF-UK), the Australian Council on Health care Standards (ACHS), the Scottish Clinical Indicators, the Canadian Institute for Health Information (CIHI), the Dutch National Institute for Public Health and the Environment (RIVM), the RAND Health Quality of Care Assessment Tools (QA Tools), and the German Inpatient Quality Indicators (G-IQI). All databases were searched using the keywords “refugee” or “asylum seeker” to identify potential QIs for this target group.

Furthermore, an additional manual search of grey literature with “Google Scholar” was conducted in June 2018. For this search the terms “quality indicator” AND “refugee” OR “asylum seeker” were used. Additionally, we scrutinized reference lists of included studies and relevant reviews identified through the search. Additionally, we conducted a review by searching PubMed, the Cochrane Library and Web of Science, using “quality indicator” and “refugee” as medical subject headings (MeSH)-terms and as text words in June 2018. The search strategy for PubMed was: (“Refugees” [Mesh] OR “refugees” [All Fields]) AND (“Quality Indicators, Health Care” [Mesh] OR “indicators” [All Fields]). The Mesh term refugee included following terms: Refugee, Asylum Seekers; Asylum Seeker; Seekers, Asylum.

The search strategy for web of science was: (refugee* OR asyl* seek*) AND (indicator).

Moreover, we cross checked the reference lists of the publications. If publications contained QIs from other indicator sets, we included the original publication of the mentioned indicator set and excluded the secondary source.

This systematic review was independently performed by two reviewers (KH, DW), who conducted the literature search and review following the PRISMA guidelines [10]. These two independent reviewers screened titles and abstracts initially for potential relevance. If the abstract matched the inclusion criteria, the full article was obtained and reviewed. After selection of potentially relevant articles, full reports were obtained and assessed for inclusion and exclusion criteria. Any disagreement on the eligibility of studies was resolved through discussion to reach consensus or, if required, by involving a third experienced review author.

Overall, the search strategy was defined by the principles of a systematic search and implied free-text keywords and Mesh terms by two reviewers who were well experienced in conducting Systematic Reviews. No medical librarian was consulted.

### Inclusion criteria and screening procedure

Publications were included if the following inclusion criteria were fulfilled:

- QIs were reported
- QIs were developed for the target group ‘refugees and asylum seekers’
- Primary source of QI
- Published in English, French or German

Quantitative and qualitative research was considered. There was no restriction of time or place, all studies from 1980 to 2018 were included in the systematic review. We included both clinical indicators and indicators for practice management. According to the key characteristics of an ideal indicator by Mainz [9] the described indicators had to be specific and measurable with the numerator and denominator principles. In a first step for full-text review, all publications were included that described QIs for refugees and asylum seekers and extracted these indicators. In a second step, all publications were excluded that identified QIs for refugees but did not have a reference to health care. The final step was to classify health care indicators based on the dimensions put forward by Donabedian [8]. These dimensions were structure, process and outcome quality. Two authors (KH and DW) independently read and extracted the data from each study included. In cases of disagreement or discrepancies, we involved a third review author (JS) to reach consensus.

**Fig. 1:**
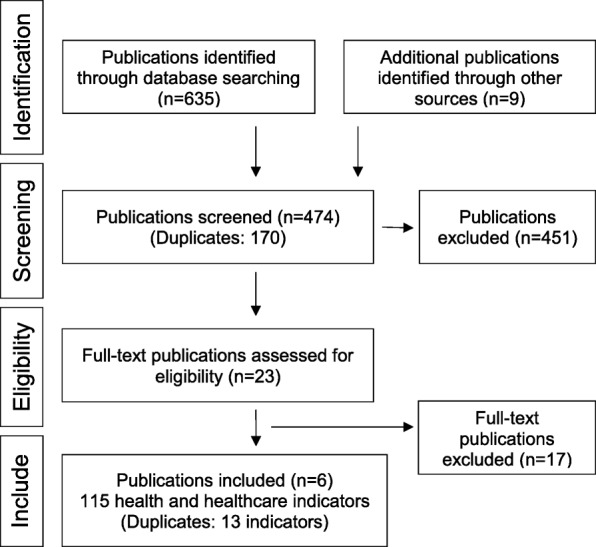
PRISMA flow chart

An overview of the literature identification and selection is presented in the PRISMA flow chart in Fig. 1.

We charted the following data from the included studies: bibliographic details such as author/source, year of publication, title, and included indicators, especially health care indicators.

## Results

In the QI databases, no indicators could be found that specifically related to refugees or were established for this target group. Using the keywords “refugee” and “asylum seekers” did not provide any results.

The review of PubMed, Web of Science and grey literature, and publications of reference lists and grey literature revealed 644 papers. The removal of duplicates left 474 papers of which 9 publications were excluded because of publication language, and 23 were eligible for a full-text review [11–33]. Most represented indicators were indicators of integration [11–13, 17], indicators of education [11–14, 17] and indicators regarding health care [11–16]. Other studies contained indicators relevant to single topics: indicators of acculturation [19], indicators of cultural participation [20], indicators of refugee placement [21], indicators with a focus on “youth in refugee camps” [22], indicators for assessing infant and child feeding practices [23], and indicators for malnutrition [24].

Publications including indicators from other primary references [25–28] and containing indicators not especially developed for refugees but applicable to this target group [29, 30] were excluded. All 17 publications [17–33] that were not relevant to health care, were excluded. Finally, 115 QIs related to health care for refugees were identified within 6 publications [11–16].

### Descriptive analysis

We found 115 indicators in 6 publications that were applicable to health care for refugees. These included 33 indicators concerning structural quality, 26 indicators concerning process quality and 51 indicators concerning outcome quality. Four indicators related to both process and structural quality and one indicator related to both outcome and process quality. Please see Table 1 for details.

**Table 1:** Overview of the number of indicators related to health and health care of refugees

Source	Title	Year	Ref.	Topics	Donabedian framework (number of indicators)
Sphere Project	The Sphere Project	2007	15	Health care services Reproductive health Health status	Process = 15 Structure = 13 Outcome = 7 Structure and Process = 3
United Nations High Commissioner for Refugees	Inter-agency Field Manual on Reproductive Health in Humanitarian Settings	2010	16	Reproductive health	Process = 7 Structure = 8 Outcome = 31
United Nations High Commissioner for Refugees	Practical Guide to the Systematic Use of STANDARDS & INDICATORS in UNHCR Operations	2006	14	Health status	Process = 2 Structure = 8 Outcome = 8
Home Office Development and Practice Reports: Indicators of Integration	Indicators of Integration	2004	12	Health care services	Process = 2 Structure = 2 Outcome = 3 Outcome and Process = 1 Structure and Process = 1
OECD	Indicators of Immigrant Integration	2015	11	Health status	Outcome = 2
UNHCR	Refugee Integration and The Use Of Indicators: Evidence From Central Europe	2013	13	Health care services	Structure = 2

*UNHCR* United Nations High Commissioner for Refugees, *OECD* Organisation for Economic Co-operation and Development, *e.g.* for example, *Ref.* Reference number

The indicators covered three thematic domains: “reproductive health”, “health care service”, and “health status”. “Reproductive health” was assessed by 58 indicators for family planning, maternal and newborn health and HIV/AIDS. “Health care service” included 46 indicators describing access to health care and health management. “Health status” included 11 indicators such as birth rates, mortality rates, and diseases. All health care indicators were listed and sorted according to these three topic domains. If indicators could be assigned to several topics, they were only listed in the predominant category.

### Reproductive health

The reproductive health indicators found in the literature covered various thematic domains. There were preventive indicators such as “number of condoms distributed per person per month” [14] and “condom use” [11], indicators regarding pregnancy or maternal and child health and some indicators regarding HIV/AIDS. The topic of reproductive health was the largest group of indicators identified within our review. Additionally, UNHCR focused on this domain in their publications [14, 16], and 8 indicators from the Sphere Project [15] were relevant to this topic.

Within this topic, there were 11 indicators for structural quality, 14 indicators regarding process quality and 32 outcome indicators. All indicators regarding reproductive health for refugees are listed in Table 2.

**Table 2:** Indicators of the topic reproductive health

Source	Donabedian framework	Indicator
Sphere (2007) [15]	Structure	There are at least four health facilities with BEmOC and newborn care/500,000 population.
Sphere (2007) [15]	Structure	There is at least one health facility with CEmOC and newborn care/500,000 population.
UNHCR (2010) [16]	Structure	Coverage of Supplies for Standard Precautions
UNHCR (2010) [16]	Structure	Coverage of HIV Rapid Tests for Safe Blood Transfusion
UNHCR (2010) [16]	Structure	Coverage of Clean Delivery Kits
UNHCR (2010) [16]	Structure	Availability of clinical management of rape survivors
UNHCR (2010) [16]	Structure	Contraceptive supply
UNHCR (2010) [16]	Structure	EmOC services availability
UNHCR (2010) [16]	Structure	STI/RTI management skills of service providers
UNHCR (2010) [16]	Structure	STI/RTI case management
UNHCR (2006) [14]	Structure	Number of condoms distributed per person per month
UNHCR (2010) [16]	Process	Timing of PEP provision
UNHCR (2010) [16]	Process	Timing of emergency contraception (EC) provision
UNHCR (2010) [16]	Process	Timing of STI prophylaxis
Sphere (2007) [15]	Process	All pregnant women in their third trimester have received clean delivery kits.
Sphere (2007) [15]	Process	People most at risk of exposure to HIV are targeted with an HIV prevention program.
Sphere (2007) [15]	Process	Pregnant women known to be HIV positive have received ARV drugs for PMTCT.
Sphere (2007) [15]	Process	100% of transfused blood is screened for transfusion-transmissible infections including HIV.
Sphere (2007) [15]	Process	Individuals potentially exposed to HIV (occupational exposure in health care settings and non-occupational exposure) have received PEP within 72 h of an incident.
UNHCR (2010) [16]	Process	Investigation of maternal deaths
UNHCR (2010) [16]	Process	EmOC services utilization
UNHCR (2010) [16]	Process	Abortion services performed with appropriate technology
UNHCR (2010) [16]	Process	Awareness of legal indications for termination of pregnancy
UNHCR (2006) [14]	Process	Have stocks of condoms run out for more than a week?
UNHCR (2006) [14]	Process	Are there any specific interventions directed at refugees/foreseen in the HIV/AIDS national strategic plan?
Home Office (2004) [12]	Outcome/Process	Immunization, antenatal care and cervical and breast screening (coverage compared with general population)
Sphere (2007) [15]	Outcome	The proportion of deliveries by caesarean section is not less than 5% or more than 15%
UNHCR (2010) [16]	Outcome	Number of Reported Rape Cases
UNHCR (2010) [16]	Outcome	Condom Distribution Rate
UNHCR (2010) [16]	Outcome	Incidence of STD in young people
UNHCR (2010) [16]	Outcome	Proportion of STI among those under 18 years
UNHCR (2010) [16]	Outcome	Proportion of births among those under 18 years
UNHCR (2010) [16]	Outcome	Condom use among young people
UNHCR (2010) [16]	Outcome	Contraceptive prevalence (CP)
UNHCR (2010) [16]	Outcome	Community knowledge concerning family planning (FP)
UNHCR (2010) [16]	Outcome	Coverage of FP counseling
UNHCR (2010) [16]	Outcome	Neonatal mortality rate
UNHCR (2010) [16]	Outcome	Proportion of low birth weight
UNHCR (2010) [16]	Outcome	Stillbirth rate
UNHCR (2010) [16]	Outcome	Complete antenatal care
UNHCR (2010) [16]	Outcome	Coverage of syphilis screening
UNHCR (2010) [16]	Outcome	Tetanus vaccination coverage
UNHCR (2010) [16]	Outcome	EmOC needs met
UNHCR (2010) [16]	Outcome	Percentage of births assisted by a skilled attendant
UNHCR (2010) [16]	Outcome	Coverage of postpartum care
UNHCR (2010) [16]	Outcome	Percentage of deliveries by Caesarean section, by administrative unit
UNHCR (2010) [16]	Outcome	Direct obstetric case fatality rate
UNHCR (2010) [16]	Outcome	Coverage of post-abortion contraception
UNHCR (2010) [16]	Outcome	Coverage of induced abortion
UNHCR (2010) [16]	Outcome	Number of cases of sexual violence reported to health services
UNHCR (2010) [16]	Outcome	Incidence of genital ulcer disease
UNHCR (2010) [16]	Outcome	Incidence of male urethral discharge
UNHCR (2010) [16]	Outcome	Quality of blood donation screening
UNHCR (2010) [16]	Outcome	VCT post-test counselling and result
UNHCR (2010) [16]	Outcome	PMTCT coverage
UNHCR (2010) [16]	Outcome	PMTCT post-test counselling and result
UNHCR (2010) [16]	Outcome	Coverage of ARV in PMTCT programs
UNHCR (2010) [16]	Outcome	Condom use

*BEmOC* Basic Emergency Obstetric Care, *CEmOC* Comprehensive Emergency Obstetric Care Services, *EmOC* Emergency Obstetric Care Services, *STI/RTI* sexually transmitted infections/ reproductive tract infections, *PEP* postexposure prophylaxis to prevent HIV transmission, *ARV* antiretrovirals, *PMTCT* prevention of mother-to-child transmission, *STD* sexually transmitted diseases, *VCT* voluntary counselling and testing

### Health care services

Within this thematic domain there were 46 indicators that focused on various topics such as access to health care for refugees, training of staff and processes necessary for safe individual and public health. Most indicators in this group were from the Sphere Project (26 indicators), which defines minimum standards for disaster-affected populations [17]. Within this topic there were 22 indicators for structural quality, 12 indicators regarding process quality and 8 outcome indicators.

All indicators regarding health care services for refugees are listed in Table 3.

**Table 3:** Indicators for the topic health care services

Source	Donabedian framework	Indicator
Home Office (2004) [12]	Structure/Process	Strategies identifiable at health authority/board level for addressing priority health needs among refugee populations
Sphere (2007) [15]	Structure/Process	No health facility is out of stock of selected essential medicines and tracer products for more than one week
Sphere (2007) [15]	Structure/Process	A written outbreak investigation and response plan is available or developed at the beginning of a disaster response.
Sphere (2007) [15]	Structure/Process	All primary health care facilities have clear standard operating procedures for referrals of patients with NCDs to secondary and tertiary care facilities.
Home Office (2004) [12]	Structure	Proportion of refugees registered with a General Practitioner (compared with general population)
Home Office (2004) [12]	Structure	The number of refugee doctors and nurses joining professional registers
Sphere (2007) [15]	Structure	There are an adequate number of health facilities to meet the essential health needs of all the disaster-affected population:
		- one basic health unit/10,000 population members (basic health units are primary health care facilities where general health services are offered),
		- one health center/50,000 people,
		- one district or rural hospital/250,000 people,
		- > 10 inpatient and maternity beds/10,000 people
Sphere (2007) [15]	Structure	Utilization rates at health facilities are 2–4 new consultations/person/year among the disaster-affected population and > 1 new consultations/person/year among rural and dispersed populations
Sphere (2007) [15]	Structure	There are:
		- at least 22 qualified health workers (medical doctors, nurses and midwifes)/10,000 population
		- at least one medical doctor/50,000 population,
		- at least one qualified nurse/10,000 population,
		- at least one midwife/10,000 population.
Sphere (2007) [15]	Structure	There is at least one Community Health Worker (CHW)/1000 population, one supervisor/10 home visitors and one senior supervisor.
Sphere (2007) [15]	Structure	Clinicians are not required to consult more than 50 patients a day consistently. If this threshold is regularly exceeded, additional clinical staff are recruited.
Sphere (2007) [15]	Structure	Primary health care services are provided to the disaster-affected population free of charge at all government and non-governmental organization facilities for the duration of the disaster response.
Sphere (2007) [15]	Structure	All health facilities have trained staff, sufficient supplies and equipment for clinical management of rape survivor services based on national or WHO protocols.
Sphere (2007) [15]	Structure	All primary health care facilities have antimicrobials to provide syndromic management to patients presenting with symptoms of an STI.
Sphere (2007) [15]	Structure	All health facilities have trained staff and systems for the management of multiple casualties.
Sphere (2007) [15]	Structure	All health facilities have trained staff and systems for the management of mental health problems.
Sphere (2007) [15]	Structure	All primary health care facilities have adequate medication for continuation of treatment of individuals with NCDs who were receiving treatment before the emergency.
UNHCR (2006) [14]	Structure	Do returnees have access to emergency and primary health care services without discrimination?
UNHCR (2006) [14]	Structure	Will there be a possibility for returnee to continue ART in returnee area?
UNHCR (2006) [14]	Structure	Number of persons per primary health care facility
UNHCR (2006) [14]	Structure	Annual no. of consultations at primary health care facilities per person
UNHCR (2006) [14]	Structure	Percentage of live births attended by skilled personnel (excl. TBAs)
UNHCR (2006) [14]	Structure	Do asylum-seekers/refugees have access to antiretroviral therapy from any source, if available in hosting community?
UNHCR (2006) [14]	Structure	Do asylum-seekers/refugees have access to primary health care services?
UNHCR (2013) [13]	Structure	Health Insurance Requirement
UNHCR (2013) [13]	Structure	Access to health care
Home Office (2004) [12]	Process	Refugee involvement in Patient Advisory & Liaison Services and similar initiatives
Home Office (2004) [12]	Process	Patient information available in culturally appropriate form regarding service entitlements, provision and relevant health risks.
Sphere (2007) [15]	Process	All health facilities and agencies regularly provide a HIS report within 48 h of the end of the reporting period to the lead agency.
Sphere (2007) [15]	Process	All health facilities and agencies report cases of epidemic-prone diseases within 24 h of onset of illness.
Sphere (2007) [15]	Process	The lead agency produces a regular overall health information report, including analysis and interpretation of epidemiological data, as well as a report on the coverage and utilization of the health services.
Sphere (2007) [15]	Process	The lead agency has developed a health sector response strategy document to prioritize interventions and define the role of the lead and partner agencies at the onset of an emergency response.
Sphere (2007) [15]	Process	Standardized case management protocols for the diagnosis and treatment of common infectious diseases are readily available and consistently used.
Sphere (2007) [15]	Process	Health agencies report suspected outbreaks to the next appropriate level within the health system within 24 h of detection.
Sphere (2007) [15]	Process	The lead health agency initiates investigation of reported cases of epidemic prone diseases within 48 h of notification.
Sphere (2007) [15]	Process	All children under 5 years old presenting with malaria have received effective antimalarial treatment within 24 h of onset of their symptoms.
Sphere (2007) [15]	Process	All children under 5 years of age presenting with diarrhea have received both oral rehydration salts (ORS) and zinc supplementation.
Sphere (2007) [15]	Process	All children under 5 years of age presenting with pneumonia have received appropriate antibiotics.
Home Office (2004) [12]	Outcome	Utilization rates of specialized services (e.g., antenatal care, mental health services, chiropody services, NHD Direct, etc.) by refugees (compared with general population)
Home Office (2004) [12]	Outcome	Refugees reported satisfaction with service provision.
OECD (2015) [11]	Outcome	Health care
Sphere (2007) [15]	Outcome	Upon completion of measles vaccination campaign:
		- at least 95% of children aged 6 months to 15 years have received measles vaccination;
		- at least 95% of children aged 6–59 months have received an appropriate dose of Vitamin A.
Sphere (2007) [15]	Outcome	Once routine EPI services have been re-established, at least 90% of children aged 12 months have had three doses of DPT (diphtheria, pertussis and tetanus), which is the proxy indicator for fully immunized children.
UNHCR (2006) [14]	Outcome	Measles vaccination coverage rate
UNHCR (2006) [14]	Outcome	Percentage of female members in asylum seeker/refugee representative bodies
UNHCR (2006) [14]	Outcome	Percentage of needs met for sanitary materials

*STI* sexually transmitted infection(s), *NCD* noncommunicable diseases, *ART* antiretroviral therapy, *TBA* traditional birth attendant(s), *EPI* Expanded Program on Immunization

### Health status

Within this group, there were 11 outcome indicators. They covered topics including “self-reported health status” [11] and incidence, mortality and birth rates [12, 14, 15]. All indicators regarding the health status of refugees are listed in Table 4.

**Table 4:** Indicators for the topic health status

Source	Donabedian framework	Indicator
Home Office (2004) [12]	Outcome	Morbidity and mortality rates compared with the general population
OECD (2015) [11]	Outcome	Self-reported health status
Sphere (2007) [15]	Outcome	The crude mortality rate (CMR) is maintained at, or reduced to, less than double the baseline rate documented for the population prior to the disaster.
Sphere (2007) [15]	Outcome	The under-5 mortality rate (U5MR) is maintained at, or reduced to, less than double the baseline rate documented for the population prior to the disaster.
Sphere (2007) [15]	Outcome	Incidence of major communicable diseases relevant to the context are stable (not increasing).
Sphere (2007) [15]	Outcome	Case fatality rates (CFRs) are maintained below acceptable levels:
		- cholera – 1% or lower
		- Shigella dysentery – 1% or lower
		- typhoid – 1% or lower
		- meningococcal meningitis – varies, 5–15%
		- malaria – varies, aim for < 5% in severely ill malaria patients
		- measles – varies, 2–21% reported in conflict-affected settings, aim for < 5%
UNHCR (2006) [14]	Outcome	Infant Mortality Rate (< 1 year) (returnees and non-returnees)
UNHCR (2006) [14]	Outcome	Child Mortality Rate (< 5 years) (returnees and non-returnees)
UNHCR (2006) [14]	Outcome	Crude Mortality Rate (returnees and non-returnees)
UNHCR (2006) [14]	Outcome	Percentage of newborn children with low birth weight (< 2500 g.) (weighed within 72 h)
UNHCR (2006) [14]	Outcome	Crude Birth Rate (annual)

Most of the indicators were outcome indicators (*n* = 51; 44.35%), structural quality was represented with 33 indicators (28.7%) and the smallest group were indicators of process quality (*n* = 26; 22.61%). Four indicators (3.48%) addressed both process and structural quality and one indicator addressed both outcome and process quality.

## Discussion

This systematic review shows evidence concerning QIs for health care of refugees and asylum seekers. Different databases were used and 115 indicators that related to health care of refugees were identified. These different indicators could be sorted into three topics, “reproductive health”, “health services” and “health status” including a categorisation into the Donabedian quality dimensions: process, structure or outcome of care. Most indicators were outcome indicators and focused on mortality and morbidity; process indicators represented the smallest group of indicators. Most of the indicators address items concerning reproductive health and maternal and child health. There are many indicators focusing on these topics due to a marked need for addressing highly prevalent conditions that should be addressed in future research. Access to high-quality reproductive health services including appropriate emergency obstetrics can drastically reduce the number of women who die during or after childbirth, ensuring that mothers and their children enjoy a healthy life. UNHCR applied the principle that reproductive health care should be offered to all refugee women [16]. Quality reproductive health services require that organizations, programs and providers use appropriate technology, have trained staff, and ensure accessible services and respectful care.

Although WHO and UNHCR have highlighted the importance of reproductive health in refugee situations, a systematic review about refugee health status shows no study regarding physical health of women during pregnancy and childbirth. The authors identify a priority need for research in this context [34].

The second largest group of indicators observed in this review are indicators concerning the topic of health care services, such as access to health care. Access to health care is a critical determinant of survival in refugee situations such as disasters. The right to health can be assured only if the health care providers responsible for the health system are well trained and comply with professional standards [15]. The organisation of health care for refugees and access to health care present a challenge for the host countries. Refugees often have no regular access to health care and they struggle with restricted access to health care in their host countries [35]. There should be standards for refugee health care access similar to the Sphere-Standards for disaster-affected populations [15]. Moreover, the provision of health care services for refugees and asylum seekers presents also a challenge for the health care providers. A systematic review shows that health care providers struggle not only with the diverse cultural beliefs and language differences but also with limited institutional capacities which additionally restrict the access to health care [36].

The third topic, indicators for health status, only contains outcome indicators such as morbidity and mortality which are connected to the indicators of reproductive health and health services. The quality of life in the country of origin, the migration process and the conditions in the host country could influence the health outcomes of refugees. Refugees may be more vulnerable to certain diseases or mental disorders than people without such experiences. The migration experience itself could create stress which could influence the health outcomes of migrants in different ways depending on the socio-economic and health conditions in the country of origin [11].

As complement to our systematic review a recently published evidence report concludes that there is a lack of common strategies for health care management of refugees and asylum seekers [3]. Owing to different legal frameworks in the host countries, no general conclusion about the accessibility and quality of health care delivery can be adopted [3]. It can be assumed that generic QIs could overcome this barrier and help to optimize and improve the health care of refugees and asylum seekers. A further review shows that different guidelines for migrant health care are available that range from disease specific to generic guidelines for health care delivery which could have an impact on quality of health care [37]. A systematic use of such guidelines, especially of the developed QIs, in the health care process of this population group is essential to ensure a high-quality of health care.

The main strength of our systematic review was the presentation of numerous and diverse areas in which QIs for refugee care were developed. The search strategy was defined by the principles of a systematic search and implied free-text keywords and Mesh terms by two reviewers who were well experienced in conducting Systematic Reviews. No medical librarian was consulted.

However, we only included publications written in English, French or German. Moreover, because of the clear defined search strategy it could be that some institutes on provincial level in different countries like the Institute of Clinical Evaluative Sciences in Ontario, Canada showed no QI for health care of refugees. Therefore, there might be a selection bias in our findings.

## Conclusions

It can be concluded that most indicators stress outcome. It can be assumed that an effective process within health care services supports high-quality of health care and should be the focus of further studies. QIs are an important measurement tool for the documentation and improvement of health care. Further research needs to address explicitly measurable QIs to learn more about health care for refugees and asylum seekers. Moreover, it can be assumed that a smaller number of indicators can be better implemented in health care of refugees. Therefore, the next step would be the reduction and prioritisation of these 115 indicators e.g. based on the RAND/UCLA Method [38].

## Data Availability

The datasets used and analyzed during the current study are available from the corresponding author upon reasonable request.

## Abbreviations

ACHS: Australian Council on Health care Standards
AHRQ: Agency for Health care Research and Quality
ART: Antiretroviral therapy
ARV: Antiretrovirals
BEmOC: Basic Emergency Obstetric Care
CEmOC: Comprehensive emergency obstetric care services
CIHI: Canadian Institute for Health Information
e.g.: Exempli gratia/ for example
EmOC: Emergency obstetric care services
EPI: Expanded program on immunization
G-IQI: German inpatient quality indicators
NCD: Noncommunicable diseases
OECD: Organisation for economic co-operation and development
PEP: Postexposure prophylaxis to prevent HIV transmission
PMTCT: Prevention of mother-to child transmission
QA Tools: RAND Health Quality of Care Assessment Tools
QI: Quality indicators
QOF-UK: UK’s Quality and Outcomes Framework
RIVM: Dutch National Institute for Public Health and the Environment
RTI: Reproductive tract infections
STD: Sexually transmitted diseases
STI: Sexually transmitted infection(s)
TBA: Traditional birth attendant(s)
UNHCR: United Nations High Commissioner for Refugees
VCT: Voluntary counselling and testing
WHO: World Health Organization
